# Supplementary material for: Dynamics of Anti-S IgG Antibodies Titers after the Second Dose of COVID-19 Vaccines in the Manual and Craft Worker Population of Qatar
Source: Vaccines (Basel). 2023 Feb 21;11(3):496. doi: 10.3390/vaccines11030496 (PMC10058349; doi:10.3390/vaccines11030496)
Supplement: Supplementary file 1 [file vaccines-11-00496-s001.zip › vaccines-2024993-supplementary.pdf]

Annexure 1

Table S1. Characteristics of participants who had anti-S IgG antibodies, by anti-NC IgG status.

Variables	Anti-NC IgG absent	Anti-NC IgG present
**N**	265	29
Age (years), median (IQR)	37.1 (31.6, 44.9)	35.0 (26.7, 39.8)
**Nationality**		
Indian	29.1%	24.1%
Nepali	35.8%	44.8%
Others	34.7%	31.0%
Missing	0.4%	0.0%
**Religion**		
Hindu	10.2%	3.4%
Muslim	59.6%	62.1%
Others	27.5%	27.6%
Missing	2.6%	6.9%
**Occupation**		
Construction	37.0%	20.7%
Others	63.0%	79.3%
**Travel history (past 2 weeks)**		
No	95%	96.5%
Yes	1.5%	3.5%
Missing	3.5%	0%
Number of handwashes (past 24 hours) median (IQR)	10.0 (4.0,18.0)	10.0 (7.0,16.0)
**Vaccine group**		
Not mRNA	10.6%	37.9%
Moderna	48.3%	34.5%
Pfizer	40.4%	27.6%
None	0.8%	0.0%
Interval between two doses (days), median (IQR)	28.0 (21.0, 28.0)	28.0 (22.0, 30.0)
Duration between sample collection and last vaccine dose (days), median (IQR)	130.0 (72.0, 173.0)	117.0 (88.0, 147.0)
**IgG_spikestatus**		
Absent	1.1%	0.0%
Present	94.7%	100.0%
Test not done (Sample Rejected)	4.2%	0.0%
Anti-S IgG antibody titre (AU/mL), median (IQR)	8879.3 (3923.0, 19846.5)	10698.8 (3377.5, 21324.1)

Table S2. Hazard rate and survival time for Non-mRNA, Moderna and Pfizer vaccines.

				**Time (in months) at which 25%, 50%, and 75% of subjects dropped into the lowest Anti-S IgG quartile**		
Vaccine type	Time at risk (months)	Incidence rate	Number of subjects	25%	50%	75%
Non-mRNA	101.13	0.198	37	2.2	3.53	4.5
Moderna	566.27	0.035	139	6.37	not reached	not reached
Pfizer	577.47	0.050	118	6.33	7.63	8.47

Table S3. Cox proportional hazard regression model results.

Variables	Hazard ratio	*p*-value	95% Confidence Interval
Moderna	0.090	0.000	0.042-0.190
Pfizer	0.121	0.000	0.059- 0.249
Age (centered)	0.994	0.965	0.965-1.024
IgGNC status	0.367	0.025	0.152-0.881
Number of subjects	281		
Number of failures	67		
Total time at risk	1203.1		
LR chi2(4)	41.33	0.000	

Annexure 2: Protocols for COVID-19 Among Labour in Qatar

Environmental sampling of COVID-19

**1. Sample location information (complete new table for each sampling collection):**	
Identification number	
Date sample collected (DD/MM/YYYY)	(DD/MM/YYYY)_/_/_<br>Time:
Room temperature at time of sampling (°C)	
Room humidity at time of sampling (%)	
Location of room in which sample was collected	□ Participant's bedroom<br>□ Participant's bathroom<br>□ Entry routing<br>□ Other
When was the room last cleaned?	(DD/MM/YYYY)___/___/______<br>Time:
When was the room last disinfected?	(DD/MM/YYYY)___/___/______<br>Time:

**2. Sampling information :**	
If yes, were multiple swabs taken?	□ Yes □ No □ Unknown
What storage medium was used?	□ Viral transport medium<br>□ Tryzol<br>□ RNAlater<br>□ Other:

**3. Storage and transport information :**	
When were the samples stored at the laboratory?	(DD/MM/YYYY)___/___/______<br>Time:

How were the samples stored at the laboratory?	□ 4°C<br>□ -20°C<br>□ -80°C<br>□ Other:
When were the samples transported to the laboratory?	(DD/MM/YYYY)___/___/______<br><br>Time:
How were the samples transported to the laboratory?	□ 4°C<br>□ -20°C<br>□ -80°C<br>□ Other:

Epidemiological and clinical information

**Patient identification number**	
**1. Current Status**	□ Alive □ Dead

**2. Data Collector Information**	
**Name of data collector**	
Data collector Institution	
Data collector telephone number	
Mobile number	
Email	
Form completion date (DD/MM/YYYY)	____/___/_
Date of interview with informant (DD/MM/YYYY)	____/___/_

**3. COVID-19 patient information**	
**First name**	
Surname	
Sex	□ Male □ Female □ Not known
Date of birth (DD/MM/YYYY)	__/_/_
Telephone (mobile) number	
Country of residence	
Nationality	
Ethnicity (optional)	
Responsible Health Centre	
Nursery/School/College if appropriate Work/ Stay home etc	
Have you travelled within the last 14 days domestically?	□ Yes □ No □ Unknown<br><br>If Yes, dates of travel (DD/MM/YYYY):<br>__/__/___ to____/_/__<br><br>Regions: Cities visited:
Have you travelled within the last 14 days internationally?	□ Yes □ No □ Unknown<br><br>If Yes, dates of travel (DD/MM/YYYY):<br>__/__/___ to____/_/__<br><br>Countries visited: Cities visited:
In the past 14 days, have you had contact with a anyone with suspect or confirmed COVID-19 infection?	□ Yes □ No □ Unknown<br><br>If Yes, dates of last contact (DD/MM/YYYY):<br>__/_/_

4a. Primary case symptoms from onset of illness	
**Date of first symptom onset* (DD/MM/YYYY)**	___/___/____ □ Asymptomatic □ Unknown
Fever (≥38 °C) or history of fever*	□ Yes □ No □ Unknown If yes, specify maximum temperature from onset of illness:
Date of first health facility visit (including traditional care)* (DD/MM/YYYY)	____/_/__ □ NA □ Unknown
Total number of visits to health facilities since onset of illness	
Total number of health facilities visited since onset of illness	□ NA □ Unknown Specify:
**4b. Respiratory symptoms**	
Sore throat*	□ Yes □ No □ Unknown If Yes, date (DD/MM/YYYY):_/_/_
Cough*	□ Yes □ No □ Unknown If Yes, date (DD/MM/YYYY):_/_/_
Runny nose*	□ Yes □ No □ Unknown
Shortness of breath*	□ Yes □ No □ Unknown If Yes, date (DD/MM/YYYY):_/_/_
**4c. Other symptoms**	
Chills	□ Yes □ No □ Unknown
Vomiting	□ Yes □ No □ Unknown
Nausea	□ Yes □ No □ Unknown
Diarrhoea	□ Yes □ No □ Unknown
Headache	□ Yes □ No □ Unknown
Neurological signs If Yes, specify	□ Yes □ No □ Unknown
Rash	□ Yes □ No □ Unknown
Conjunctivitis	□ Yes □ No □ Unknown
Muscle ache	□ Yes □ No □ Unknown
Joint ache	□ Yes □ No □ Unknown
Loss of appetite	□ Yes □ No □ Unknown
Nose bleed	□ Yes □ No □ Unknown
Fatigue	□ Yes □ No □ Unknown
General malaise	□ Yes □ No □ Unknown
Seizures	□ Yes □ No □ Unknown
Altered consciousness	□ Yes □ No □ Unknown
Other symptoms	□ Yes □ No □ Unknown If yes, specify:

5. Primary case pre-existing condition(s)	
**Obesity**	□ Yes □ No □ Unknown
Cancer	□ Yes □ No □ Unknown
Diabetes	□ Yes □ No □ Unknown
HIV/other immune deficiency	□ Yes □ No □ Unknown
Heart disease	□ Yes □ No □ Unknown
Asthma (requiring medication)	□ Yes □ No □ Unknown
Chronic lung disease (non-asthma)	□ Yes □ No □ Unknown
Chronic liver disease	□ Yes □ No □ Unknown
Chronic haematological disorder	□ Yes □ No □ Unknown
Pregnancy	□ Yes □ No □ Unknown If yes, specify trimester: □ First □ Second □ Third □ NA Estimated delivery date (DD/MM/YYYY) ___/_/_
Chronic kidney disease	□ Yes □ No □ Unknown
Chronic neurological impairment/disease	□ Yes □ No □ Unknown
Organ or bone marrow recipient	□ Yes □ No □ Unknown
Other pre-existing condition(s)	□ Yes □ No □ Unknown If yes, specify:
